# Winning a Won Game: Caffeine Panacea for Obesity Syndemic

**DOI:** 10.2174/157015910791233213

**Published:** 2010-06

**Authors:** M Myslobodsky, A Eldan

**Affiliations:** 1 Howard University Graduate School (Washington, DC); Clinical Brain Disorders Branch, NIMH/National Institutes of Health (Bethesda, USA); 2 WideMed Ltd., Israel

**Keywords:** Caffeine targets, obesity, reactive oxygen species, networks, polypharmacy.

## Abstract

Over the past decades, chronic sleep reduction and a concurrent development of obesity have been recognized as a common problem in the industrialized world. Among its numerous untoward effects, there is a possibility that insomnia is also a major contributor to obesity. This attribution poses a problem for caffeine, an inexpensive, “natural” agent that is purported to improve a number of conditions and is often indicated in a long-term pharmacotherapy in the context of weight management. The present study used the “common target” approach by exploring the tentative shared molecular networks of insomnia and adiposity. It discusses caffeine targets beyond those associated with adenosine signaling machinery, phosphodiesterases, and calcium release channels. Here, we provide a view suggesting that caffeine could exert some of its effects by acting on several signaling complexes composed of HIF-1α/VEGF/IL-8 along with NO, TNF-α, IL1, and GHRH, among others. Although the relevance of these targets to the reported therapeutic effects of caffeine has remained difficult to assess, the utilization of caffeine efficacies and potencies recommend its repurposing for development of novel therapeutic approaches. Among indications mentioned, are neuroprotective, nootropic, antioxidant, proliferative, anti-fibrotic, and anti-angiogenic that appear under a variety of dissimilar diagnostic labels comorbid with obesity. In the absence of safe and efficacious antiobesity agents, caffeine remains an attractive adjuvant.

## INTRODUCTION

Obesity prevalence has visibly increased over the recent decades to the point where its spread was dramatized by a comparison with an epidemic. With a long list of its comorbidities, it has become a burden at the individual, familial, and societal levels. The word ‘obesity’ itself is a recognized synecdoche, a figure of speech in which the whole may stand for a part; a particular cause (e.g., hyperphagic obesity), appearance problem, local expansion of adiposity (e.g. visceral obesity), or “obesity-related diseases” [1], for which only individually tailored medical solutions could be entertained. 

In what might appear as an effort of an ‘environmental reductionism’, a progressive decrease in nightly sleep time is now suggested to be a catalyst of obesity [2-5]. Chronic sleep shortage that in the last decades afflicts at least one-third of the population in the industrialized world [6] has become a novel bastion of the obesogenic “environment” and is now listed among the contributors to the visceral form of obesity that underlies the metabolic syndrome [7-9]. Insomnia is a recalcitrant therapeutic problem. Therefore, with numerous other paths to obesity [10], designing a highly innovative drug that produces all the expected therapeutic effects by targeting a particular ‘common mechanism’ [11] seems unrealistic. In efforts to reduce costs, health professionals are turning to novel information on the mode of action of inexpensive old drugs. In a sobering revelation of Allen Roses, then with GlaxoSmithKline (cited in [12]), fewer than half of the patients prescribed some of the most expensive drugs actually derived any benefit from them. With this authoritative assessment of a leader in applied pharmacogenetics, our choice is to review here the benefits of an old and inexpensive agent, such as caffeine that is an integral part of a normal everyday routine. 

## BACK TO AN OLD PARADIGM

Adiposity, much as insomnia(s) might be conceived of as “pseudopathology”. This paleopathological term in Crawford’s language [13] means a quasi-normal phenomenon in the sense that both are useful adaptations that are no longer essential and could be (but not necessarily are) harmful. Because of a significant degree of overlap that exists between normal and aberrant, pseudopathology represents a recalcitrant research problem. Unwittingly, insomnia-obesity coupling creates a significant therapeutic problem because caffeine, a member of the methylxanthine family of drugs (1,3,7-trimethylxanthine) and the oldest of the socially endorsed addictive psychostimulants, is also employed to combat food craving and weight gain. The figures of the United States National Coffee Association (cited in [14]) indicate that over 50% of adult Americans drink coffee every day, mostly as a “caffeine fix” or as a “pick-me-up” morning ritual. With a daily load of at least 4 mg/kg per regular coffee drinker in the US, and twice that in Scandinavia [15], caffeine consumption was rightfully named an ‘epidemic’, that is suggestively parallel to that of obesity. The flagrant irony here is that a reputed somnolytic is used to curb weight gain that could be attributed inter alia to insomnia. 

A recent review [16] restated a familiar admonition that caffeine, even when taken as early as 16-17 hours prior to sleep, could induce subtle and physiologically meaningful changes in the sleep EEG, in particular in the self-rated caffeine-sensitive subjects. In reality, we do not know who may be vulnerable to weight gain following sleep deprivation and whether those categorized as being “sensitive to caffeine” in one domain would manifest a similar responsiveness to it in other conditions. Sleep is the latest addition to the list of the obesogenic factors. While its social plausibility is appealing, the role of insomnia in obesity is not compelling. Insomnia or better said, insomnias appear in dissimilar profiles and severity and are confounded by the standard of living, age, overall health status [17, 18], as well as by numerous methodological problems of their analysis [9]. A recent study by Lauderdale *et al*. [19] found no evidence that sleep duration influences gaining weight over the span of 5 years. Adenosine is an endogenous somnogen, and the central effect of caffeine is believed to act *via *the antagonism of adenosine receptors (A1, A2A, A2B, and A3, subsequently as ADORA1, ADORA2A, ADORA2B, and ADORA3). Ever since the influential review by Snyder and Sklar [20], caffeine has been perceived as a “magic” nonselective antagonist of ADORA1 and ADORA2A. That view had steered much of the neurobiological research and was used as a thread to guide further studies [21, 22]. The question is whether the somnolytic effects disqualify caffeine as a potential anti-obesity medicine. Not necessarily. 

## MINING CAFFEINE TARGETS

Caffeine affects diverse bodily tasks and can thus be affected by multiple physiologic variables that are almost impossible to take into account. In order to reduce the complexity of the expected target display, we employed a commercially available engine for drug target identification and recording their interaction, *Ingenuity Pathway Analysis* (IPA) [23]. Fig. (**1**) provides a summary of ‘caffeine neighborhood’ compiled using IPA.8. In it, caffeine molecule appears as a hub that establishes connections by crossing the boundaries of other intrinsic modules, thereby creating a temporary ‘parasitic’ unit. Such drug hubs were termed elsewhere ‘pharmodules’ [24]. The point of this distinction is in the fleeting character of such sub-networks that gradually disappear with a half-life determined by the drug elimination or that of its metabolites. 

Predictably, caffeine node is connected to the adenosine receptors, as well as several molecules, *via *the shortest paths that do not instruct us about the orthogonal somnolytic and weight gain effects (Fig. (**2**)). One might expect that its somnolytic effects implicate diverse neurotransmitter systems, which agonists and antagonists are on the current agenda of the pharmaceutical industry [25]. Among them, only tachykinin precursor 1 (TAC1) may be of interest. TAC1 is a member of tachykinin family of peptides that is widely distributed in the central, peripheral, and enteric nervous systems of many species. It was implicated in modulation of many behavioral and physiological functions including emotional behaviors. Recently, TAC1 was also recognized as a novel antiobesity target. A selective antagonist of TAC1 receptor, (*CJ012,255; *Pfizer, New York, NY) given parenterally was reported to prevent weight gain in lean mice placed for 2 weeks on high-fat, whereas similar treatment of genetically obese mice resulted in weight loss and reduced adiposity, along with an improved insulin sensitivity [26]. The role of TAC1 in sleep is still uncertain. It may cause central alertness and insomnia [27]. Yet when microinjected bilaterally into a sleep-promoting region in the preoptic area, it rather appears to promote sleep [28]. Neurokinin 1 receptor antagonist casopitant is under development by GlaxoSmithKline (*GSK-679769*) as an antidepressant, anxiolytic, and hypnotic/sedative [25]. 

The effect of caffeine *via *TAC1 may be more complex because it increases activation of tumor necrosis factor-α (TNF-α). However, *in vitro* study of human adipose tissue obtained after abdominal dermolipectomy showed a strong and dose-dependent down-regulation of TNF-α expression in both adipocyte and stromavascular fraction cells, whereas interleukin-6 (IL-6) was only down regulated in the latter cells [29]. That is an interesting result, particularly for adiposity developed in the elderly, as IL-6 gene knockouts were noticed to manifest ‘mature-onset’ obesity [30]. Thus, by decreasing the expression of proinflammatory cytokine TNF-α, caffeine could quench the inflammatory potential of adipose tissue [29, 31, 32]. 

Several other cytokines affected by caffeine could influence the EEG parameters, albeit after local neocortical application (reviewed in [33]). For example, growth hormone (GH)-releasing hormone (GHRH) applied in small doses to the surface of the rat somatosensory cortex, decreased the EEG delta waves on that side, while higher doses enhanced the delta power. Similar to its microinjection into the preoptic anterior hypothalamus, GHRH enhanced the EEG delta power and duration of the non-REM periods in a state dependent manner, i.e., it occurred during non-REM state but not during REM sleep [34]. Caffeine in concentrations that are relevant to human consumption had little or no effect on IL-1β, whereas TNF-α production was suppressed in all individuals studied [31]. 

Other molecules of functional relevance could only be revealed *via *IPA path analysis with an extra or a ‘linker’ node. Such hitherto unexplored targets are hypocretin/orexin neuropeptides that are distributed to the majority of cell groups that regulate behavioral arousal. Consequently, their depletion might lead to sleep aberration akin to narcolepsy. One study, found the adenosine-mediated inhibition of the firing of hypocretin/orexin neurons without a change in their membrane potential. The inhibitory effect of adenosine was dose dependent and mediated by ADORA1 [35]. Caffeine did not activate this system. However, some clusters of ADORA1 heteromerize with ADORA2A receptors into the ADORA1-ADORA2A heteromer. This entity could lead to opposite read-outs of chemical signals in the system. For example, in this way, caffeine produces the opposite effects on glutamate release at low and high concentrations of synaptic adenosine ([36, 37] and references therein). 

An unexpected second-tier entity in the network was melatonin (MEL). Caffeine was reported to induce a prolonged nocturnal rise in pineal MEL, an endogenous regulator of circadian rhythms of several biological functions, including sleep-wake cycles in humans and other mammals [38]. In a study by Härter *et al*. [39], caffeine (200 mg) co-administered with oral MEL increased its bioavailability by 142% on average. This may be a clinically relevant augmentation, given that in humans MEL bioavailability is poor [40]. In another study in humans, evening caffeine at a dose corresponding to two ordinary cups of coffee was reported to augment the nocturnal serum MEL levels [41]. 

MEL is able to affect the regulation of body mass and energy balance *via *leptin (LEP). MEL-supplemented mice had significantly higher plasma LEP levels than control mice [42]. LEP is an anorectic hormone produced in adipose cells. Treatment with LEP was shown to significantly decrease food intake, adiposity, glucose and insulin plasma contents, and blunt the treadmill running-induced elevation in plasma levels of corticosterone [43]. MEL receptors are widely distributed in diverse tissues, such as epithelial cells, liver, intestine, kidney, immune cells, including adipocytes, i.e., the cells that synthesize LEP [44], such that the sedative effects of LEP may be as relevant as the ingestion control implemented *via *MEL. Regardless of the sleep-promoting efficacy of MEL, it may be an interesting, and hitherto unexplored, anti-obesity agent. Preclinically, MEL proved to reduce body mass in diet-induced obesity in Sprague Dawley rats [45, 46]. Again in preclinical studies, MEL as well as its novel agonist NEU-P11, inhibited body weight gains, deposits of abdominal fat and metabolic profiles in obese rats with no influence on food intake [47]. 

The circulating LEP plasma values are increased at night in rodents and in humans [48, 49] although the strength of the effect, and even its presence, varies as a function of glucose and insulin levels and body mass. Male *ob/ob* mice, a genetic model of impaired LEP production that results in severe obesity along with symptoms of metabolic dysregulation, showed sleep fragmentation and the elevated number of arousals from sleep [50]. Acute LEP administration altered sleep architecture in normally fed rats. It decreased REM sleep by about 30% and somewhat increased non-REM sleep time (by 13%), mostly in the delta-frequency band (a common EEG marker of sleep intensity). These effects, too, were coupled with energy status and were absent in conditions of food deprivation [49]. 

Recent studies imposing nocturnal-life style on adult volunteer students reported a simultaneous decrease in MEL and LEP levels [51]. The style of diet was virtually identical to the pattern of eating imposed during the month of Ramadan. In this Moslem holiday, food intake is restricted to the night hours, thereby causing sleep delay. Ramadan fasting diminished a peak in nocturnal MEL consequent to modifications in sleep schedule [52]. A study conducted by the same group five years later exposed significant shifts of 5 h 30 min in peak and trough serum LEP levels on the twenty-third day of Ramadan [53]. 

The simultaneous attenuation of the nocturnal increase in LEP and MEL is reminiscent of that observed in patients with night-eating syndrome [54]. A delayed food intake caused disrupted circadian patterns of several hormones by causing phase delays of LEP and insulin (INS), as well as in the circadian MEL rhythm. The circulating levels of ghrelin, the primary orexigenic hormone was phase advanced by 5.2 h. Their amplitudes were also blunted, save for the increased thyroid-stimulating hormone (TSH) amplitude [54]. The LEP-MEL coupling is a complex process that is apparently determined by a number of metabolic participants [51]. *In vitro* analysis, using isolated rat adipocytes incubated with MEL (1 nM) and INS, did not affect LEP expression when administered alone, whereas administered together they have increased it by 120%. A synthetic glucocorticoid dexamethasone showed meager effects on LEP. However, when added simultaneously with MEL, LEP release was increased by a robust 250% [44].

A recent study found no significant differences in LEP levels in chronic insomnia patients as compared to healthy control men [55]. However, control individuals were matched for body weight with insomnia patients so that the relevance of this finding to insomnia-induced obesity is questionable. As though emphasizing this contention, stage 4 in normal subjects was shorter when compared to those with insomnia. 

## CAFFEINE IN OBESITY SYNDEMIC

Obesity scatters an array of molecules that in different combinations are implicated in inflammation, angiogenesis, cellular proliferation, and cellular injury [56]. Among the latter are reactive oxygen species (ROS), a group of O^2^− molecules in different states of oxidation or reduction, as well as further-derived reactive species, such as hydrogen peroxide and hypochlorous acid. The etiology of several human ailments is attributed to cellular damage induced by ROS. 

ROS are important components in the vicious cycle triggered by adiposity because they are believed to modulate the adipogenesis. The term describes the course of mesenchymal stem cells (preadipocytes) commitment, their proliferation, differentiation into mature fat (adipocytes), and their hypertrophy. More specifically, ROS accelerate the rate of evolution of preadipocytes into the mature lipid-laden adipocytes, which are the predominant source of pro-inflammatory adipokines [57]. By comparison, smaller young adipocytes capable of proliferation act as ‘metabolic buffers’ that avidly absorb fatty acids in the postprandial period [58-60]. A failure to store excess lipid in adipocytes *via *hyperplasia will inevitably channel fatty acids into non-adipose tissues [61, 62], thereby fueling low-grade inflammation and consequently leading to metabolic syndrome [63, 64]. As an effective scavenger of hydroxyl radicals and singlet oxygen, caffeine may be helpful when the endogenous antioxidants, such as glutathione and the enzyme superoxide dismutase are depleted [65]. 

The potential therapeutic role of caffeine in obesity may be in controlling angiogenesis. Angiogenesis is the process of formation of new vessels from preexisting structures of endothelial and vascular supporting cells (reviewed in [66, 67]). The mechanisms by which new vascular network is created has yet to be worked out. Adipose tissue is capable of self-maintenance by its intrinsic angiogenic potency [68] that assures supply of oxygen and nutrients and a removal of waste products. A seminal hypothesis of tumor growth proposed by Folkman [69] prompted the idea that visceral fat expands in a manner of malignant cells, by recruiting neovascularization in order to mitigate hypoxia. Growth of solid tumors is a multistage process that begins with an avascular stage followed by a vascular phase, which depends on the induction of angiogenic factors VEGF and IL-8. 

Adenosine appears capable of a massive stimulation of angiogenesis by acting as a “survival hormone” essential for adaptation during hypoxia, neuronal trauma, response to different kinds of stressors, as well as cellular workload [70, 71]. It rises 100-fold during periods of oxygen depletion and ischemia [72] and conspires with hypoxia in rapidly deploying neoangiogenesis. The most fascinating aspect of this process is that it triggers an evolutionarily conserved pathway governed by the hypoxia-induced factor-1 (HIF-1) [73]. The presence of HIF-1 is a key catalyst and regulator of the delivery of oxygen, because it induces several angiogenic factors identified thus far, such as vascular endothelial growth factors (VEGFs), interleukin-8 (IL-8) and others([74] for review and references therein). The VEGF family in humans currently comprises several members, namely, VEGF-A, VEGF-B, VEGF-C, VEGF-D, erythropoietin (EPO), placental growth factor (PGF) and a novel regulatory factor, fibroblast growth factors (FGF) [75]. Some of them are illustrated in Fig. (**2**). VEGF-A is a particularly interesting isoform of the VEGF family [76] that has received attention for its increase of the vascularization needed for the expansion of adipose tissue [77]. It creates a destabilized network with disorganized, leaky, immature, and unstable vessels [74]. That is how omentum-induced angiogenesis could be facilitated [78] under hypoxic conditions. Caffeine antagonizes the up-regulation of factor-1 (HIF-1) along with VEGF, and IL-8. 

Anti-angiogenesis agents are considered a viable treatment option in malignancies [79]. Likewise, the VEGF system may represent a profitable target for the pharmacological treatment of obesity. For example, bevacizumab, a recombinant humanized monoclonal antibody to VEGF was tested recently in preclinical animal models. It caused loss of weight and anorexia as a side effect of therapy [80]. It cannot be recommended off-label for obesity in view of the fact that it may induce hypertension. Although the mechanisms of elevated blood pressure in patients treated with anti-angiogenesis agents is not well understood, mechanistically, it may be attributed to the existing deficit in the form of capillary dropout (rarefaction) [81-83]. Patients with hypertension have reduced microvascular density, with some evidence supporting a primary role for rarefaction in causing hypertension. Although rarefaction may be present in normotensive persons, revealingly, they have a family history of hypertension [84]. Compared with lean subjects, overweight/obese individuals appear to have 44% lower capillary density and 58% lower VEGF, suggesting that rarefaction could drive obesity *via *hypoxia and inflammation [85]. 

Caffeine might be a better choice for targeting the signaling cascade of angiogenesis. It was suggested to be a promising therapeutic agent with a direct impact on neovascularization of human tumors *via *VEGF inhibition [86]. Even oral administration of green tea (*Camellia sinensis*) has been noticed to inhibit the formation and growth of several tumor types in animal models [87]. VEGF up-regulation was abrogated by caffeine. It reduced by an impressive 46% the neovascularization in a mouse ischemic hind limb model [88]. To what extent caffeine is a functionally relevant agent to affect transmural pressure and vasomotion in mechanically altered leaky vessels is difficult to predict [89]. However, it promises to curb mechanically triggered neointimal hyperplasia. In this way, it can mitigate a major cause of morbidity following stent deployment in patients with coronary artery disease [90]. This is an important effect, as healthy individuals with mild obesity, ischemia, insomnia, and inflammation may be exposed to hypoxia during a long-term stay at moderate altitude (> 1700 m), e.g. when skiing. Fortunately, in skiers and hikers only EPO was elevated, whereas VEGF levels remained unchanged. In keeping with this result, post-altitude examinations at 7-10 days and 6-7 weeks showed that body fat has actually decreased during the study [91]. 

More devastating episodes of hypoxia are experienced by patients afflicted by obstructive sleep apnea [56, 92, 93]. Caffeine is not considered for therapeutic intervention in apnea, perhaps in fear of further increasing sympathetic outflow, blood pressure, and ultimately increasing the frequency of awakenings. But perhaps, apnea may not always have to be aggressively treated. In a study of comorbidities associated with moderate sleep apnea in the elderly, Lavie and Lavie [94] obtained reduced mortality risk, indicating that cellular stress of apnea could bring to the pathophysiological forefront some molecular circuits responsible for ‘hormetic’ or ‘preconditioning’ effects [95, 96]. This ‘apnea paradox’ cautions that the taxonomically driven drug indications may need to be revisited. More than 60% of patients with obstructive sleep apnea represent a heterogeneous category of individuals who are devoid of the expected nocturnal fall in blood pressure (defined as ‘non-dippers’) *independent of obesity* [97], so that they may be afflicted by a peculiar vascular dysregulation. Blunted reduction of nocturnal blood pressure (operationally defined as fall of systolic and diastolic blood pressure <10%) was associated with higher levels of high-sensitivity C-reactive protein [98], a marker of inflammation associated with increased cardiovascular morbidity. Would they benefit from ingesting coffee? A study of 982 diabetic and 1,058 non diabetic women without cardiovascular disease from the Nurses’ Health Study may provide a tentative answer. These women who drank ≥ 4 cups of coffee a day obtained favorable metabolic effects including decreased insulin resistance, decreased incidence of type 2 diabetes, and lower levels of markers of inflammation. The effect is believed to partly operate through increased serum adiponectin levels [99]. 

The sufferers of chronic sleep apnea commonly manifest polycythemia, a state of abnormally increased circulating red blood cells that is associated with the significantly higher serum levels of VEGF [100]. Given that VEGF plays a key role in the angiogenic response, caffeine may be an agent of choice in polycythemia, as well. Oddly, those with polycythemic state are noticed to ingest increased quantities of caffeine-containing beverages [101]. The only hypothetical impediment of caffeine ingestion might be in its abrupt discontinuation with no support of lifestyle changes in individuals with intrinsically hypovascular adiposity. It is possible to speculate that similar to what happens in secondarily avascular and hypoxic tumors [66], a wave of angiogenesis prompted by VEGF accumulation may rebound in weight gain. 

A higher average daily caffeine intake was noticed to improve the cognitive performance in patients with moderate/severe sleep apnea [102]. This is in keeping with the ability of caffeine to strengthen a long-term memory formation by activating cortical neural oscillators *via *glutamate release from nerve terminals [103], as reflected in hippocampal gamma oscillations. The latter are an EEG frequency range associated with the cognitive functions that is suppressed during hypoxia [104]. In neuropsychiatry, obesity is discussed among modifiable factors of age-associated memory impairment that may also herald Alzheimer’s dementia [105] when caffeine may be counted among other memory enhancers. In a recent study in rats given 25 mg/kg pentylenetetrazol (a seizure-producing agent known to cause retrograde amnesia) or ethanol, caffeine (5 mg/kg) canceled ethanol-induced retrograde amnesia in a new odor-recognition test [106]. It is of interest that a combination of a phosphodiesterase-5 inhibitor and an adenosine ADORA2A antagonist, mimicking two major mechanisms of action of caffeine, prevented the memory impairment, though neither drug alone had such effect. 

As an inhibitor of phosphodiesterase-5, a key enzyme in the biochemical pathway mediating erection, caffeine may inspire a practitioner to try it off-label as benign aid in erectile dysfunctions, inasmuch as these are a common complication of obesity and associated with it diabetes mellitus and insulin-resistance [107]. Preclinically, an 8-week administration of caffeine improved the erectile function in diabetic rats by inhibiting phosphodiesterases and thus up-regulating cavernous intracellular cyclic guanosine monophosphate [108]. Caffeine may be of benefit in other androgen dysfunctions associated with obesity, such as oligozoospermia and a reduced spermatozoa motility count [109]. The latter were noticed to be associated with increased BMI [110]. 

The account may continue into peripheral neuropathology such as sensorineural hearing loss that may also result from abnormalities of the VIII cranial nerve. Among other factors, obesity along with chronic hyperglycemia may be implicated in the early sensorineural hearing loss [111]. Preclinically, coffee consumption was shown to facilitate recovery from diabetes-induced auditory neuropathy in diabetic mice [112]. Finally, psoriasis, a chronic inflammatory skin disease, as well as psoriatic arthritis, are common among obesity comorbidities [113]. A double-blind, placebo-controlled study proved that topical application of 10% caffeine appears to be an effective, safe and inexpensive treatment for the stable plaque psoriasis [114]. 

In sum, this admittedly selected sample suggests that caffeine might be a useful agent to handle a range of conditions associated with obesity, if only we knew how to use it more profitably outside of the “Starbuck” coffeehouses. That leads to the next point. 

## “REPURPOSING” OF CAFFEINE

An interesting feature of caffeine is its ability to enhance the efficacy of co-administered agents without compromising their safety. Caffeine co-administration with aspirin was noticed to cause long-lasting hyperactivity [115]. Combining caffeine (10 mg/kg) with a low amount of alcohol (‘caffeinol’ [116]) appears to reduce the cortical infarct volume and improves recovery following stroke. In a model of rotating rodent (‘hemiparkinsonian mice’), daily co-administration of caffeine to L-DOPA produced enhanced rotational behavior, i.e., a promising feature to think of the non-dopaminergic augmentation in the treatment of Parkinson's disease [117]. 

Adding caffeine to ephedrine has produced a noticeable weight reduction, whereas either drug given alone yielded effects similar to those achieved in the placebo group [118]. A cross-sectional population-based telephone survey of 9,403 adults revealed that an estimated 20.6% of women and 9.7% of man used a weight-loss supplement containing caffeine (with other stimulants such as ephedra) [119, 120]. Patients with hypothalamic obesity who had been steadily gaining weight benefited clinically from a regimen of caffeine and ephedrine [121]. In one trial, a “caffeine+” design was used with nicotine in order to amplify anorectic effects of the latter [122]. In addition, it has a range of time effects that regulate the body fat stores from millisecond that are relevant for changing satiety, to setting a stage for a lasting reduction in food intake *via *sympathetic activity control and plasticity changes *via *glutamate [123]. Caffeine pairing with novel flavors appears to raise their subjective predilection as compared to liking of the novel flavors paired with a placebo [124], thereby suggesting that, oddly enough, caffeine may enhance the common underlying neurobiological and psychological mechanisms of desire. It is long known that caffeine has adjuvant pain-relieving properties in some cases, even though it is not clear why analgesia may not appear in others [125, 126].

These co-administered drugs are so diverse that clearly no common molecular denominator for their combinations is currently in sight. For example, preclinical studies in the rat inoculated with osteosarcoma exposed a striking enhancement by caffeine of the cytocidal effects of platinum-based anticancer drug cisplatin. The extent of tumor inhibition was closely correlated with the average plasma concentration of caffeine [127]. Likewise, adding caffeine to standard chemotherapy enhanced the antitumor response rates in patients with osteosarcoma. On the median follow-up period of 72 months, it extended event-free survival in all patients to 76%, whereas an overall survival reached 100% [128]. Caffeine has also been reported to modulate directly or indirectly the effect of antitumor agents, so as to greatly sensitize tumor cells to genotoxic stress and thus aid in cancer chemoprevention [86]. Chronic exposure to caffeine prior to the appearance of the palpable mammary tumors significantly reduced both the tumor burden and the metastatic colonization [129]. 

All considered, caffeine has an adequate record of safety in long-term ingestion [130, 131] (see, however, [132]); for adopting it for a diversity of “orphan” conditions based on emerging needs. This transgression of nosological indications is a novel trend in the current climate of dwindling health care resources and rising costs of drug development [133]. Most commonly, it appears in the form of a pragmatic ad-hoc off-label prescription on physician’s initiative that ultimately, may be institutionalized as a drug “repurposing” or drug “repositioning” [134, 135]. A review by Ashburn and Thor [136] makes an admirable exhibit of several appealing Cinderella stories illustrating an ‘indication switch’. One of them is a miraculous comeback of thalidomide that after its tragic fall has now numerous indications. Among thalidomide’s parallel biographies is a discovery of its anti-angiogenic properties that has made it a candidate drug in oncology. As mentioned above, caffeine could acquire a parallel resume by improving the clinical outcome in patients with malignancy [86, 129]. Moreover, it taps into a deeper need to reconsider an antiquated practice (and legislation) based on nosological indications in favor of ‘fixing’ a particular pathophysiological aberration that is shared by several disease entities. 

However, before caffeine attracts attention of the pharmaceutical and biotech companies, its appeal for “repurposing” has to be prepared by network analysis. Visualizing the drug-target landscapes would pave the way for future designs. By detailing molecular targets and links that otherwise might be overlooked, caffeine pharmodule could evolve into a decision-making tool by providing effective filters for considering the role of age, gender, race, and comorbidities [137] when designing a promiscuous agent for a particular disease state. An important feature of drug interactions in networks is that their connections are ‘weighted’ because formally, a number (‘weight’) can be assigned to each edge (Fig. (**2**); insert). In order to illustrate its meaning, a triad NF-κB-TAC1-TNF is isolated on the right along with all the interactions between each of its members labeled next to the edges. As analyzed elsewhere [138], each node of the set is a participant in more than one biochemical reaction (e.g., activation, inhibition, biochemical modification, protein-protein interaction, transcription, translocation, and other). Consequently, each two neighbors of “functional motifs” may be active in a variety of ways at different conditions because its edges indicate that they could become functionally engaged on an unpredictable timetable. Their functional proximity is determined by the common roles in a particular ‘pharmodule’, rather than the presence of certain structural or pharmacophoric features. That is, they belong to the mutual ‘biospace’, regardless of whether they share binding properties and thus are also united in the same ‘chemospace’ [139]. Therefore, the orientation of the arcs cannot reflect the course of information flow in network, i.e., it cannot confidently predict the sequence of responses to a drug in pharmodule. Although IPA provides an efficient and powerful means for mining of drug action and disease mechanism relationships it has yet to include adequate formalism for such predictions to be modeled. 

## ON WINNING A WON GAME: THERAPEUTIC IMPLICATIONS AND CAVEATS

Management of obesity is torn between two opposite tendencies: pharmacotherapy and lifestyle changes. Due to its modest efficacy and side effects, the pharmacotherapy of obesity is hoped to effect a cure chiefly when it is weaved into environmental changes. Caffeine is an example of an ‘ergogenic/metabolic perspective’ slanted in the direction of reducing adiposity in people with a “low-energy-output phenotype” [140]. 

In a symmetric concession, therapeutic virtues of the personal responsibility in controlling appetite and lifestyle are believed to be boosted by the future antiobesity drugs [141]. These drugs would not control an epidemic of weight gain, and realistically, practicing physicians are busy dealing with a catalog of diverse co-morbidities of obesity [142]. In this regard, obesity is truly a “syndemic” rather than an epidemic. The term “syndemic” was coined in the early 90ies by the anthropologist Merrill Singer (reviewed in [143]) to emphasize the role of environmental factors in the context of their synergistic relations with some major afflictions. The ‘upgrading’ of an epidemic into the syndemic implies the need for novel drug targets that are capable of handling numerous chronic comorbidities along with those causing the ‘core’ affliction. 

Caffeine might evolve into such a drug. Its broad-spectrum therapeutic potential includes lipolytic, neuroprotective, nootropic, antioxidant, proliferative, anti-fibrotic, anti-angiogenic, and anti-diabetic effects [70]. Why then is formal position in the pharmacotherapy so insignificant. Perhaps, to paraphrase the famous line of Frank Marshall, “The hardest thing in pharmacology is to win a won game”. By prevailing in its social acceptance as a common drink, caffeine has lost in prestige and its potential medicinal appeal has declined; its pharmacological research has become peripheral and academically unrewarding. Nonetheless, a demand for the ‘maximization of a drug value’ in the current pharmaceutical market [11, 135], seems to make it an attractive candidate for a “repurposing” program. 

There are some problems on this path. Caffeine could modulate clinical phenotypes by interacting with several signal transduction pathways and becoming their temporary member. As network analysis revealed (Fig. (**2**)), some tentative caffeine targets cluster as though to suggest the need to influence the functionality of the group at once and in a predictable sequence. For example, HIF-1 and VEGF are activated *via *ADORA3A, whereas IL-8 is recruited *via *the ADORA2B subtype. That permitted to specify the set of HIF-1α/VEGF/IL-8 as a collective target in the context of tumor hypoxia for the development of the new antitumor drugs [144]. Inhibition of the extracellular signal-regulated kinase 1/2 (ERK1/2), p38, and Akt might be seen as still another complex caffeine target [144]. Further, by inhibition of the transcription nuclear factor kappa B (NF-κB), caffeine could be linked to a range of processes, from the initiation of tumorigenesis to cell survival, suppression of cell proliferation, and plasticity [145-147]. In a similar way, induction in the NF-κB protein by IL1 and TNF-α stimulates the production of NO and adenosine [33]. It is possible that insomnia increases the adenosine levels in bodily tissues *via *NF-κB and along with NO, TNF-α, IL1, and GHRH modulates the duration and intensity of non-REM sleep [148]. 

In caffeine repurposing, a persistent recommendation to limit caffeine ingestion by those who may be at risk of reduced bone mineral density [149], particularly in post-menopausal women may need to be addressed. How important is it to curb caffeine ingestion in this age group has yet to be determined, but here is a tentative example. In the past, fluoride was prescribed precisely for the fear of fractures associated with the reduction of bone volume. A Cochrane review showed that fluoride did improve the bone density as expected, but ‘paradoxically’ rather increased the risk of a non-vertebral fracture [150]. An explanation of the outcome may not be so paradoxical, because the increased bone density was obtained at the price of its diminished elasticity. Interestingly, bones of aged rats on a diet supplemented with caffeine (2 mg/100 g body weight), showed inferior mechanical properties except for the modulus of elasticity [151].

Recently, the caffeine consumption was found to be significantly and independently associated with suicidal acts in patients with bipolar disorder [152]. It is possibly the only agent to cause a withdrawal headache under placebo-controlled double-blind conditions [153]. Its indications may need to be monitored even in obesity cases. Consider a small fat deposit around the heart (‘epicardial adipose tissue’). It was recently suggested to have health benefits by modulating the state of coronary arteries and myocardium through paracrine and direct secretion of pro and anti-inflammatory adipokines, including increased adiponectin production ([154] and references therein). It may well act as a local anti-inflammatory signal by inhibiting the NF-κB protein and consequently, IL-6 and TNF-α expression. In addition, as Iacobellis’ group proposed, epicardial adipocytes may sponge excessively high circulating levels of fatty acids and thus serve as a local energy source for myocardium in times of high demand. 

Some aspects of the caffeine pharmacological profile are consistent with its antagonistic or perhaps, reverse agonistic effects at GABA_A _and/or benzodiazepine sites [155]. Unexpectedly, caffeine increased [^35^S] TBPS binding (a standard test of increased ability to generate Cl^-^ currents). The latter is known to be directly related to the increased GABAergic neurotransmission [155]. This promiscuity of caffeine should be kept in mind in its repurposing in view of the fact that paradoxically, its proconvulsant efficacy might antagonize the experimental wave-spike discharges [156]. A similarly suggestive effect of caffeine was obtained in a study with a developmental model of epilepsy. In the latter, repeated postnatal administration of caffeine to rats (10 and 20mg/kg s.c. daily from P7 to P11) was challenged at days P18- and P25 with metrazol, in order to induce a spike-wave activity (a common model of human absences). Caffeine showed significant anticonvulsant effects at P18, but not in 25-day-old animals. Neither the EEG nor the incidence and the pattern of minimal clonic seizures changed with added dose of the convulsant. Some younger animals in both control age groups exhibited a transition to generalized tonic-clonic seizures, yet no similar seizures were elicited in caffeine-pretreated 25-day-old animals [157]. 

To summarize, off-label prescribing is only a minor part of the program. In order to achieve a greater goal of expanding the potential of caffeine efficacy, it may need to be combined with some complementary agents into a single pill, thereby creating a drug with new therapeutic properties. Additionally, caffeine utility may be further enhanced by designing its analogues with greater selectivity for the major classes of adenosine receptors [22]. That would conceivably require some supporting pharmacokinetic, pharmacodynamic, and toxicology information for the FDA approval of a new indication. How soon this hope actually bears fruit remains to be seen. 

## Figures and Tables

**Fig. (1):**
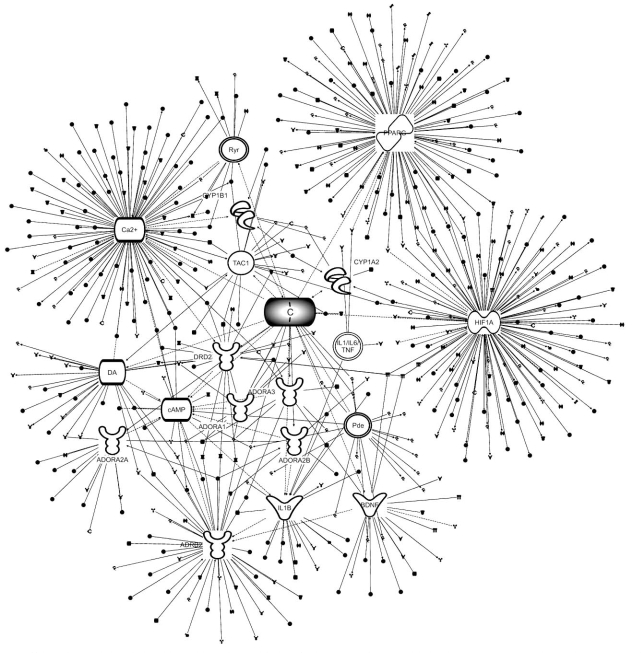
Caffeine pharmodule relationships with nodes and hubs of other networks assembled using IPA 8.0 (www.ingenuity.com). Nodes stand for any biological entity capable of functional interaction, such as genes, proteins, receptors or small molecules. The connections can be undirected or directed (arcs) that signify any relationships between the nodes listed in IPA connectivity menu (e.g. co-expression, physical interaction, phosphorylation, co-localization, etc). Each hub is connected by edges with diverse distant systems. To simplify the graph such connections and interconnections were eliminated. Abbreviations: ADORA1-adenosine A1 receptor; ADORA3-adenosine A3 receptor; ADORA2A-adenosine A2a receptor; ADORA2B-adenosine A2b receptor; ADRB2-adrenergic, beta-2 receptor; BDNF-brain-derived neurotrophic factor; cAMP-cyclic AMP; CYP1A2, CYP1B1-cytochrome P450; DA-dopamine; DRD2-dopamine receptor D2; HIF1A-hypoxia inducible factor 1, alpha subunit; IL1/IL6/TNF--IL1B-interleukin 1, beta; Pde-Phosphodiesterase; PPARG-peroxisome proliferatoractivated receptor γ; Ryr- Ryanodine receptor; TAC1-tachykinin.

**Fig. (2):**
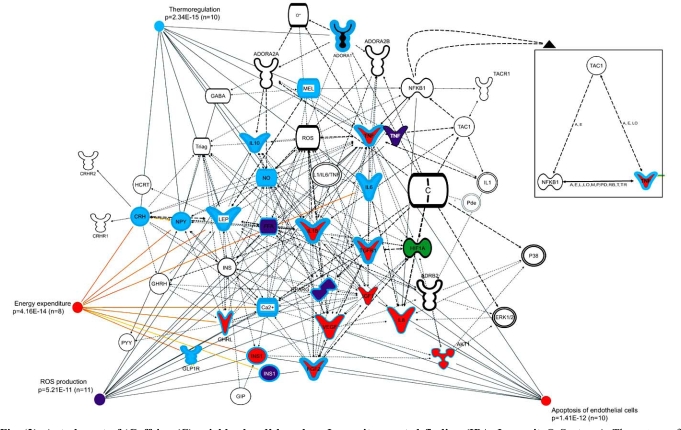
**Auto-layout of ‘Caffeine (C) neighborhood’ based on Ingenuity curated finding (IPA, Ingenuity® Systems).** The nature of interaction although available in the database or incorporated in labels of edges/arcs, was stripped from graphs to reduce the clutter, save for an example of maximal motif NF-κB-TAC1-TNF isolated on the right (see text for explanations). In the latter case, and in the rest of the nodes, self-connections were removed. The type of interaction is only identified as either ‘direct’ or ‘indirect’ (dashed lines stand for indirect interactions; solid lines indicate direct interactions). The various shapes of nodes denote the functional class of the molecules that are easily understood from their abbreviations. In order to reduce the clutter, molecular interactions that appear unrelated to the effect were trimmed. Additional molecules of interest were simply inserted by entering their terms into IPA search engine one by one or using multiple terms searches and endorsing all desirable information on connections in the ‘New pathway’ (or any available stored pathway) window via commands, such as ‘connect,’ or ‘path analysis’ based on PubMed data. Path analysis is based on network property known as node ‘betweenness’; the number of shortest paths between any chosen nodes. The latter is the smallest number of steps between any arbitrarily chosen nodes in the network selected for analysis. When there were several shortest paths between the nodes, the first one was selected as better substantiated by literature. These commands permitted to manually edit networks and in this way to create custom molecular pathways supported by the current experimental literature evidence. Color-coding depicts multiple roles that molecules may play in several signaling pathways. Arbitrarily selected are: Apoptosis of endothelial cells (p=1.41E-12); ROS production (p=5.11E-11); Energy expenditure (p=4.16E-14); and Thermoregulation (p=2.34E-15. More than four functions would make the graph more confusing in the chosen format. The probability that each biological function assigned to the set of molecules is due to chance alone was determined using Fischer’s exact test.
